# Transcriptome Dynamics in Mango Fruit Peel Reveals Mechanisms of Chilling Stress

**DOI:** 10.3389/fpls.2016.01579

**Published:** 2016-10-20

**Authors:** Velu Sivankalyani, Noa Sela, Oleg Feygenberg, Hanita Zemach, Dalia Maurer, Noam Alkan

**Affiliations:** ^1^Department of Postharvest Science of Fresh Produce, Agricultural Research Organization, Volcani CenterRishon LeZion, Israel; ^2^Department of Plant Pathology, Agricultural Research Organization, Volcani CenterRishon LeZion, Israel; ^3^Department of Plant Science, Agricultural Research Organization, Volcani CenterRishon LeZion, Israel

**Keywords:** transcriptome, mango fruit, fruit response, cold storage, chilling injury, lenticel discoloration, lipid peroxidation

## Abstract

Cold storage is considered the most effective method for prolonging fresh produce storage. However, subtropical fruit is sensitive to cold. Symptoms of chilling injury (CI) in mango include red and black spots that start from discolored lenticels and develop into pitting. The response of ‘Keitt’ mango fruit to chilling stress was monitored by transcriptomic, physiological, and microscopic analyses. Transcriptomic changes in the mango fruit peel were evaluated during optimal (12°C) and suboptimal (5°C) cold storage. Two days of chilling stress upregulated genes involved in the plant stress response, including those encoding transmembrane receptors, calcium-mediated signal transduction, NADPH oxidase, MAP kinases, and WRKYs, which can lead to cell death. Indeed, cell death was observed around the discolored lenticels after 19 days of cold storage at 5°C. Localized cell death and cuticular opening in the lumen of discolored lenticels were correlated with increased general decay during shelf-life storage, possibly due to fungal penetration. We also observed increased phenolics accumulation around the discolored lenticels, which was correlated with the biosynthesis of phenylpropanoids that were probably transported from the resin ducts. Increased lipid peroxidation was observed during CI by both the biochemical malondialdehyde method and a new non-destructive luminescent technology, correlated to upregulation of the α-linolenic acid oxidation pathway. Genes involved in sugar metabolism were also induced, possibly to maintain osmotic balance. This analysis provides an in-depth characterization of mango fruit response to chilling stress and could lead to the development of new tools, treatments and strategies to prolong cold storage of subtropical fruit.

## Introduction

Cold storage is considered one of the most effective methods for prolonging the shelf life of fresh produce. Immediate postharvest cold storage of fruit slows cellular respiration rate and metabolic processes related to ripening, thus extending fruit storage and shelf life (McGlasson et al., 1979). However, fruit such as mango (*Mangifera indica*) grown in tropical and subtropical regions are sensitive to low-temperature storage (Sivakumar et al., 2011). The climate and growth conditions during fruit development influence the sensitivity of harvested fruit to cold storage (Ferguson et al., 1999). Mature mango fruit are susceptible to CI at storage temperatures below 12°C (Mukherjee and Smock, 1958; Nair and Singh, 2003). Although consumption of mango fruit is on the rise worldwide due to its appealing taste, aroma, and nutritional value (Tharanathan et al., 2006; Sivakumar et al., 2011), CI limits the application of cold storage during transport of mango fruit from producer to consumer countries (Sivakumar et al., 2011).

Chilling injuries are physiological dysfunctions that occur in susceptible fruit stored at suboptimal, non-freezing temperatures (Lyons, 1973). These dysfunctions cause reversible primary CI and permanent secondary CI and consequently, cell death (Sevillano et al., 2009; Lukatkin et al., 2012). Chilling temperature induces various structural changes in fruit termed CI symptoms. Visible symptoms of CI in mango fruit are expressed on the peel as red and black spots, pitting or sunken lesions, peel browning, abnormal ripening, reduced aroma and flavor, and increased susceptibility to decay and poor fruit quality (Kane et al., 1982; Chaplin et al., 1991; Sivankalyani et al., 2016). The mango peel is more susceptible to CI than the pulp (Farooqi et al., 1985). Mango cultivars vary in chilling susceptibility (Farooqi et al., 1985; Phakawatmongkol et al., 2004).

Oxidative stress and an increase in reactive oxygen species (ROS) are early plant responses to chilling stress (Scandalios, 1993). Cold-signal-transduction pathways in plants are regulated through cellular influx of calcium ions (Knight et al., 1996). *Rboh* (respiratory burst oxidase homolog; NADPH oxidase) is a major player in ROS accumulation induced by calcium in response to chilling (Sagi and Fluhr, 2006; Miller et al., 2008). This oxidation plays a key role in the activation of MAP kinase (MAPK), WRKY, and downstream stress genes to cope with chilling (Thomashow, 1999).

Primary events of CI in plants are associated with peroxidation of membrane lipids, saturation of membrane fatty acids, and degradation of phospholipids (Parkin and Kuo, 1989; Lee et al., 2005). These modifications change lipid composition and membrane fluidity and cause eventual membrane impairment (Lyons, 1973). Thus, these changes disturb cellular homeostasis and lead to changes in lipid metabolism (Lyons, 1973; Kaniuga, 2008).

The mango transcriptome was recently sequenced to delineate the fruit’s response to hot-water brushing treatment (Luria et al., 2014), ripening (Dautt-Castro et al., 2015), and single-nucleotide polymorphisms (Sherman et al., 2015). However, the response mechanism to chilling stress in mango fruit has not been studied. Our aim was therefore to elucidate the molecular basis of CI and chilling response in mango fruit by evaluating the fruit peel transcriptome during postharvest cold storage. We characterized ‘Keitt’ mango fruit’s response to cold stress, which induces various physiological changes, lenticel discoloration and lipid peroxidation, and correlated them with major transcriptome changes, involving several signal-transduction and metabolic pathways.

## Materials and Methods

### Fruit and Suboptimal Temperature Storage

Mango fruit (*Mangifera indica* L. cvs. Keitt and Shelly) were obtained from a commercial storage house (Mor Hasharon, Israel) 1–2 h after harvest and transported (1 h) to the Agricultural Research Organization (Israel). Uniform, unblemished fruit weighing 424 ± 16 g were selected. To remove the fruit sap, the fruit was dipped into water after harvest; no other treatment was applied after harvest.

After harvest, six biological replicates with 10 fruits each were stored at 5, 8, 12, or 18°C for 19 days in cold-storage rooms, with a further 7 days of SL storage at 20°C. The temperature in the cold-storage room was monitored by a DAQ tool (double-strand wire logger/data acquisition control system; TMI Barak Ltd., Israel). Fruit core temperature was monitored using a MicroLite data logger (LITE5032P-EXTA; Fourier Technologies, Israel) by inserting the probe 5-cm deep into the near calyx portion of the fruit. The experiments were repeated in three consecutive seasons: 2013, 2014, and 2015, with cvs. ‘Shelly’ and ‘Keitt’ and showed similar results. Presented is the experiment with cv. Keitt in 2014.

### Evaluation of Mango Fruit Response to Cold Storage

CI symptoms in mango fruit cv. Keitt were determined by external appearance of the fruit after cold storage (5, 8, 12, or 18°C) and after 7 more days of SL storage (20°C). The severity of the external CIs—red spots, black spots, and pitting—was assessed on a relative severity index scale of 0–10 (1 representing mild CI and 10 representing severe CI, 60 evaluations per treatment). General decay and stem end rot were represented as percentage of fruit with decay in one case (six biological replicates evaluated per treatment).

### Evaluation of Ripening Parameters

Physiological parameters of mango fruit ripening: firmness, color, total soluble sugars (TSS) and titratable acidity (TA, in citric acid equivalents) were assessed at harvest, after 19 days in cold storage and after 7 days of SL. Fruit firmness (in Newton) was determined by a penetrometer (LT-Lutron FG-20KG, Indonesia) with an 11-mm probe at two points on the equatorial line of each fruit (six measurements per treatment). The mango fruit peel color was evaluated quantitatively using Chromometer CR-400/410 (Konica Minolta, Osaka, Japan) at the green side of the fruit on the equatorial line of each fruit (10 measurements/treatment). For TSS and TA determinations, 1 mL of mango pulp juice was dissolved in 40 mL double-distilled water. TSS (%) was measured with Palette Digital Refractometer PR-1 (Model DBX-55, Atago, Japan), six measurements per treatment. TA was determined as citric acid equivalent mass using an automatic titrimeter (Model 719s Titrino Metrohm Ion Analysis Ltd., Switzerland), six measurements per treatment.

### Evaluation of Lipid Peroxidation by *In vivo* Imaging System (IVIS) and Malondialdehyde (MDA) Analysis

The same cv. Keitt mango fruit was used to detect lipid peroxidation level with a preclinical IVIS (PerkinElmer, USA) and by MDA analysis. Fruit were analyzed at harvest and after 2, 7, 14, and 19 days of cold storage at 12, 8, or 5°C and a further 1 day (day 20) and 7 days (day 26) of SL at 20°C. Fruit were preadapted in complete darkness for 2 h prior to IVIS evaluation. Lipid peroxidation in fruit was detected and visualized by autoluminescence of peroxide lipids as in (Birtic et al., 2011; Sivankalyani et al., 2016), using a previously described programmed setup (Sivankalyani et al., 2016). Luminescent image data were processed and presented as total flux (W m^-2^s^-1^ per steradian) as described previously (Sivankalyani et al., 2016).

Malondialdehyde (MDA) accumulation in mango fruit peel was measured as described (Hodges et al., 1999). Mango fruit peel tissue (±2 g) was randomly collected from six fruits in each treatment with three biological replicates and two technical replicates. The MDA equivalents were calculated as described previously (Hodges et al., 1999) and expressed as nmol g^-1^ fresh weight.

### Histological Analysis

Histological analyses were performed on discolored lenticels and parts of ‘Keitt’ mango peel with CI collected 19 days after cold storage at 5 or 12°C. Tissue were fixed in FAA (10% formaldehyde, 5% acetic acid, 50% ethanol, v/v in water). Fixation was followed by an ethanol dilution series (50, 70, 90, 95, 100, and 100% × 2) and a subsequent stepwise exchange of ethanol with Histoclear (xylem substitute). Samples were embedded in paraffin and cut in a microtome (Leica RM2245, Leica Biosystems, Nussloch, Germany) into 12-μm sections in transverse and paradermal orientation. Sections were stained with safranin and fast green (Ruzin, 1999), and photographed under a light microscope (Leica-DM500, Heerbrugg, Switzerland) with an ICC50 HD camera at various magnifications (10×, 20×, and 40×).

### Scanning Electron Microscopy (SEM)

Scanning electron microscopy analysis of non-discolored and discolored lenticels was performed on peel parts of healthy and chilling-stressed ‘Keitt’ mango fruit 19 days after cold storage at 5 or 12°C. Samples were fixed in FAA then dehydrated in a graded ethanol series (50, 70, 90, 95, and 100% × 2), critical-point dried in a Quorum K850, and coated with gold palladium (Quorum SC7620 mini sputter coater). Images were taken with a JEOL JCM6000 benchtop scanning electron microscope.

### RNA Extraction, Library Preparation, and RNA-Seq

Mango fruit peel tissue (±5 g) was randomly sliced from six fruit per biological replicate at harvest and after 2, 7, and 14 days of cold storage at 5 or 12°C, each with two biological replicates. Total RNA was extracted from the peel tissue as described previously (Djami-Tchatchou and Straker, 2012). RNA quality and quantity were determined using a ND1000 UV–VIS spectrophotometer (NanoDrop Technologies Inc., USA). The RNA was treated with DNase and purified (TURBO DNA-free Kit, Ambion Life Technologies, USA). RNA integrity number >8.0 was confirmed using the Bioanalyzer 2100 (Agilent Technologies, USA). cDNA libraries were prepared for sequencing according to the manufacturer’s instructions (True Seq; Illumina Inc., USA). Libraries from two biological replicates per treatment were sequenced by the Illumina Hiseq2000 system using a 50-bp single-end RNA-Seq protocol (Nancy and Stephen Grand Israel National Center, Weizmann Institute of Science, Israel).

### Data Analysis, Annotation, and Differential Expression Analysis

The raw reads of 14 libraries were subjected to quality trimming and filtering, and adapter removal by trimmomatic software (Bolger et al., 2014). Cleaned sequences were mapped to a reference mango transcriptome (Luria et al., 2014) using bowtie2 software alignment protocol (Langmead and Salzberg, 2012). Abundance estimates were calculated for each mango transcript using the RSEM software package (Li and Dewey, 2011). Bioconductor EdgeR (Robinson et al., 2010) of the Bioconductor R packages (Gentleman et al., 2004) was used to identify differentially expressed transcripts for each biological replicate, based on the count estimates for each transcript. Transcript counts were normalized by calculating reads per kilobase per million (RPKM) (Mortazavi et al., 2008) and expressed genes were defined by considering fold change and false discovery rate (FDR) (**Supplementary Table S1**). All data analyzed in this manuscript were deposited in GenBank under accession number SRP066658.

The genes were annotated (Luria et al., 2014) by BLASTx (Altschul et al., 1990), and assigned a gene ontology (GO) term (Consortium, 2013) by combining BLASTx data and interproscan analysis (Hunter et al., 2009) by means of the BLAST2go v2.5 software pipeline (Conesa et al., 2005). GO-enrichment analysis was carried out by use of Fisher’s exact test with multiple testing correction of FDR. Transcripts that were more than fourfold differentially expressed with a FDR-corrected statistical significance smaller than 1e-5 were considered differentially expressed. The expression patterns of the transcripts at different time points were studied using cluster analysis of differentially expressed transcripts in at least one pairwise biological replicate comparison. Expression normalization was calculated using trimmed mean of *M*-values. Then, hierarchical clustering of transcripts and biological replicates was performed and clusters were extracted using hierarchical clustering based on Euclidean distance matrix (with the R scripts hclust function). Principal component analysis (PCA) and 2D hierarchical clustering were performed on normalized data using R package ‘FactomineR’ (Le et al., 2008). Transcripts of upregulated clusters were annotated with Kyoto Encyclopedia of Genes and Genomes (KEGG). Upregulated transcripts were mapped to their associated KEGG pathways.

## Results And Discussion

### Physiological Manifestation of CI in Mango

**FIGURE 1:**
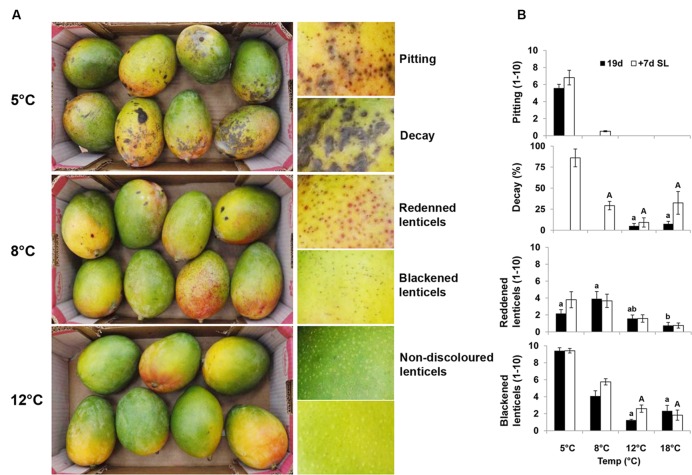
**‘Keitt’ mango fruit chilling injury (CI) symptoms and their quantification. (A)** Representative pictures of mango fruit showing CI symptoms after 19 days of cold storage at 5, 8, or 12°C. Fruit stored at 5°C show pitting and decay, at 8°C black spots and red spots, and at 12°C healthy tissue and lenticels. **(B)** Quantification of CI in mango fruit at various cold-storage temperatures (18, 12, 8, or 5°C) for 19 days (black column) and further shelf-life storage at 20°C for 7 days (white column). Red spots, black spots and pitting were evaluated on a scale of 1–10, and total decay in percentage. Data shown are mean ± SE of six biological replicates. Letters represent significant difference by one-way ANOVA.

Mango fruit is commercially stored at 10–12°C. Storage at suboptimal temperatures leads to CI and poor fruit quality. Fruit responses to cold storage of mango cvs. Shelly and Keitt were evaluated at various storage temperatures in three consecutive seasons (2013–2015). Cv. Keitt is relatively sensitive to chilling (Farooqi et al., 1985) and showed more severe CI symptoms. The results for ‘Keitt’ in season 2014 are presented here and discussed. To determine the importance of physical attributes important for fruit storage and to select conditions for transcriptome and further molecular analyses, we first examined the influence of temperature storage on external physical parameters. Red and black spots were observed on ‘Keitt’ mango fruit peel in an increasing pattern as storage temperatures declined (**Figures 1A,B**). Pitting, and black and red spots were previously observed at 5°C in cv. Tommy Atkins and characterized as CI symptoms (Pesis et al., 1997). Fruit stored at 18 and 12°C (commercial storage) showed very minor black and red spots and no pitting, with good overall fruit quality. After 15 and 19 days of storage, fruit stored at 18 and 12°C, respectively, started to ripen (**Supplementary Figure S1**). Mild decay was observed upon further storage, due to enhanced ripening (**Figure 1B**). Storage at the suboptimal temperature of 8°C led to the development of minor red and black spots (**Figures 1A,B**). Cold storage at 5°C induced the development of more black spots and pitting. Therefore, storage at 5°C showed more severe CI symptoms than storage at 8°C (**Figures 1A,B**). While fruit stored at 5°C developed fewer red spots than those stored at 8°C, fruit stored at 5°C developed more black spots and pitting. This suggests that the red spots darken to black spots under severe chilling stress, possibly due to increased accumulation of toxic phenols and their oxidation (Tamjinda et al., 1992; Grassmann et al., 2002).

To mimic SL in the market, fruit after cold storage at all temperatures were stored for an additional 7 days at 20°C. Fruit kept at 5°C showed a significant increase in decay after SL storage compared to fruit stored at higher temperatures (**Figure 1B**). Fruit stored at 18°C was over-ripe after SL storage, which also resulted in increased decay (**Figure 1B**).

Chilling stress effect on mango ripening was evaluated by standard physiological parameters, including fruit firmness, TSS, peel color change, and TA (citric acid) after 19 days of cold storage (18, 12, 8 or 5°C) and a further 7 days of SL storage (20°C). Fruit showed increased softening and decreased citric acid content with increasing storage temperature (**Supplementary Figure S1**), whereas TSS were not modified in our experiment in response to cold. Color (Hue) change values (from green to yellow) increased after cold storage at 18 and 12°C and further increased after SL storage. Fruit stored at 5 and 8°C showed delayed color change (**Supplementary Figure S1**). These results indicated that low temperature storage significantly delays fruit ripening and leads to non-uniform ripening. The delayed ripening was consistent with previous results in other cultivars (González-Aguilar et al., 2001; Wang et al., 2008).

### Mango RNA Sequence Data Analysis

To better understand the mango fruit’s response to cold stress, RNA samples were collected at harvest and after 2, 7, and 14 days of cold storage at 5 or 12°C, each with two biological replicates. A total of 222,097,481 raw reads were obtained from 14 libraries of mango peel. All data analyzed in this manuscript were deposited in GenBank under accession number SRP066658. Low-quality reads were trimmed. Clean reads were aligned to the previously published mango fruit ‘Shelly’ transcriptome (Luria et al., 2014). Overall, 57,576 transcripts with a mean length of 863 bp were identified (**Supplementary Table S1**). Transcript counts were normalized by calculating RPKM (Mortazavi et al., 2008) and expressed genes were defined by considering fold change and FDR (**Supplementary Table S1**).

**FIGURE 2:**
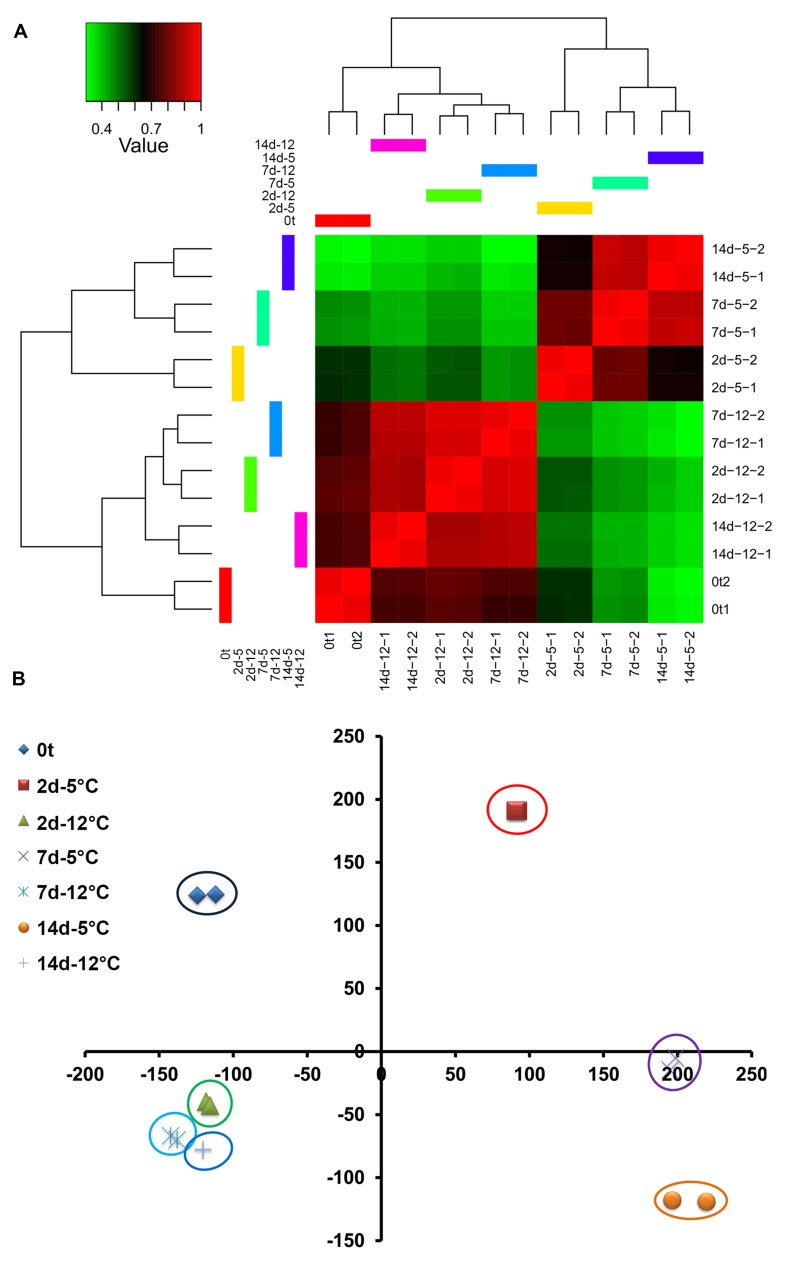
**Mango fruit global transcriptomic response to cold storage (5 and 12°C) at harvest (0t) and after 2, 7, and 14 days of cold storage. (A)** 2D hierarchical clustering, red – high correlation, green – low correlation. **(B)** Principal component analysis (PCA) at different time points and storage temperature.

Examination of 2D hierarchical clustering showed highly similar transcriptome fingerprints of fruit stored at 5 or 12°C for 2, 7, and 14 days within the same temperature treatment (**Figure 2A**). However, major differences were found between treatments. The fruit transcriptome at harvest was relatively similar to that of fruit stored at 12°C and different from that of fruit stored at 5°C (**Figure 2A**). Among the transcriptomes of fruit stored at 5°C, those at later time points (7 and 14 days) were more closely related. PCA of mango fruit’s transcriptomic response to cold strongly supported the results of the 2D hierarchical clustering, and further indicated the high transcriptome similarity between time points of fruit stored at 12°C, whereas the transcriptome of fruit stored at 5°C shifted sharply to the right after 2 days of storage (**Figure 2B**).

### Differential Expression of Genes Induced by Chilling Stress

**FIGURE 3:**
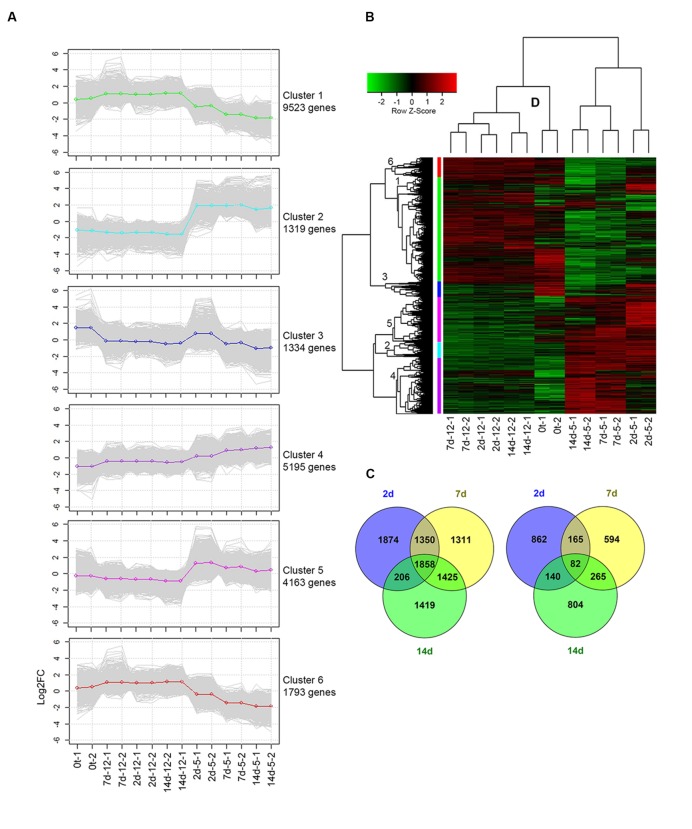
**Differential expression profile of transcripts following cold storage. (A)** Plots present the expression patterns of six clusters at different time points. Gray lines mark the various gene profiles; green, light-blue, blue, purple, pink, and red lines represent the average expression profiles of clusters 1 to 6, respectively. **(B)** Heat map diagram showing the differential expression profiles of 12,355 genes of mango fruit at four sampling times: harvest (0t) and after 2, 7, and 14 days of cold storage at two different storage temperatures (5 and 12°C) with two biological replicates. **(C)** Upregulated and **(D)** downregulated genes in response to cold storage at 5°C compared to 12°C are presented by Venn diagram at different sampling times (2, 7, and 14 days).

Hierarchical clustering and heat map analysis showed differential expression patterns of regulated transcripts [log fold change (FC) over 2 or under -2) and FDR < 0.00001] at harvest, and during cold storage at 5 or 12°C (**Figure 3**). Overall, 12,355 transcripts were regulated at 5°C compared to 12°C (FC over 2 or under -2 and FDR < 0.05). The number of upregulated transcripts (9,443) was dramatically higher than that of downregulated transcripts (2,912) at 5°C (**Figures 3C,D**). Transcripts were grouped into six different clusters according to their differential expression patterns (**Figures 3A,B**). Heat map patterns of transcripts at harvest and at 12°C were similar, while major differences were observed in comparison to storage at 5°C (**Figure 3B**). Comparative analysis of regulated transcripts showed different patterns of upregulation in clusters 2, 4, and 5 and different patterns of downregulation in clusters 1 and 6 at 5°C compared to 12°C and at harvest (**Figure 3A**).

To identify the biological reactions related to the chilling-upregulated clusters, each cluster was evaluated for its GO-enriched profile according to corrected *p*-value (FDR < 0.05) using Fisher’s exact test. Overall, 23,062 transcripts were found with GO descriptions (**Supplementary Table S1**), and 9,228 of these were differentially regulated. The overrepresented GO terms in the chilling-upregulated clusters (2, 4, and 5) were evaluated (**Supplementary Figure S2**). In cluster 2, chilling-induced genes had enriched GO terms in the biological process category related to response to abiotic stress, such as “response to temperature stimulus,” “response to oxidative stress,” “calcium ion transmembrane transport,” and more; enrichment in the molecular function category was also associated with responses to abiotic stress, such as “calcium-transporting ATPase activity,” “glutathione transferase activity,” and more (**Supplementary Figure S2**). Similarly, in cluster 5, important enriched GO terms in the biological process category were “response to salicylic acid,” “phenylalanine metabolism,” “regulation of cellular response to stress,” and “regulation of cell death” (**Supplementary Figure S2**). Overall, various GO terms related to abiotic stress were overrepresented. To better understand the functions of these overrepresented GO terms, we characterized the signaling and metabolic pathways that were upregulated during chilling.

### Activation of Chilling-Related Pathways

To characterize chilling-related pathways, 3,230 transcripts of up- and downregulated clusters were annotated according to KEGG ontology^1^. In upregulated clusters 2, 4, and 5 – 659 upregulated genes were identified with KEGG descriptions. Upregulated genes were mapped to KEGG pathways according to the *Solanum lycopersicum* database^2^. “Induction of plant stress response,” “phenylalanine and phenylpropanoid biosynthesis,” “glycerophospholipid metabolism” and “starch–sucrose–galactose metabolism” were the most significant upregulated pathways.

### Chilling Stress-Induced Plant Stress Response

**FIGURE 4:**
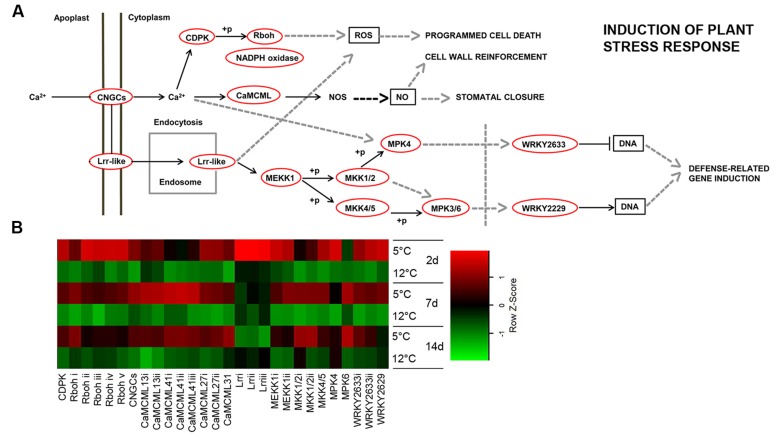
**Plant stress-response-signaling pathway induced in response to cold storage. (A)** Plant induction of stress-response-signaling pathway based on the KEGG pathway mapper. Genes that are circled in red are significantly upregulated during cold storage at 5°C. **(B)** Expression heat map of genes related to induction of the plant stress response at two different storage temperatures (5 and 12°C) at different sampling times (2, 7, and 14 days). *Z*-scores represent rescaled log fold change values. Abbreviations, transcripts identification and expression profile are described in **Supplementary Table S2**.

In mammals, specific receptors for low temperature have been identified, including the menthol receptor and a specific class of ion channel TRPA1 (Peier et al., 2002; Karashima et al., 2009). However, no such channel has been identified in plants. Our transcriptomic data showed upregulated transcripts for plant stress response at an early time point, after 2 days of cold storage at 5°C. We chose the highest ranking BLAST score as the tentative tomato or *Arabidopsis* homolog. The identified plant stress-response pathways were activated through at least two different signaling cascades of transmembrane-bound receptors: *CNGC 15II* (cyclic nucleotide-gated channel 15, comp32768) and *Lrr1*, *2* and *3* (leucine-rich repeat receptor, comp13580, comp45558, comp50036). These receptors further activated a global downstream stress response (**Figure 4**).

In plants, cold is also sensed via changes in plasma membrane fluidity (Martiniere et al., 2011), which have been suggested to lead to influx of calcium (Ca^2+^) and activation of cold-sensitive calcium channels (Carpaneto et al., 2007). Here, we show that based on transcriptomic data, a primary chilling response may be mediated by a calcium-signaling cascade. We identified an isoform of receptor protein CNGC—*CDPK* (calcium-dependent protein kinase, comp11876), and eight different isoforms of the calcium-responsive gene *CaMCML* that were upregulated at 5°C (comp21148, comp21487, comp12808, comp12808, comp28120, comp20351, comp25572, and comp27410). Moreover, five isoforms of *Rboh* (respiratory burst oxidase homolog—NADPH oxidase, comp2801, comp3011, comp31929, comp905, comp7213) were upregulated with a maximum increase at 2 days of storage at 5°C (**Figure 4**; **Supplementary Table S2**).

*MKK2*, *MPK4*, and *MPK6* have been shown to be upregulated in response to cold (Teige et al., 2004). Interestingly, *mkk2* mutation reduces the ability of *Arabidopsis* to acclimate to cold, and overexpression of *MKK2* induces many cold genes in the absence of cold (Teige et al., 2004), suggesting that *MKK2* is a positive regulator of chilling-related genes. Our mango transcriptomic results supported a role for MAPK in the cold response. Indeed, most of the MAPK cascades (*MEKK1*, comp26998, comp24341; *MKK1/2*, comp26833, comp25129; *MPK4*, comp19510; *MKK4/5*, comp14929; *MPK6*, comp2001) were upregulated in response to cold storage at 5°C (**Figure 4**; **Supplementary Table S2**). MAPK is known to activate various WRKYs, which further induce stress-related genes (Kim and Zhang, 2004). We identified two isoforms—*WRKY33* and *WRKY47*—that were upregulated in response to chilling (**Figure 4**; **Supplementary Table S2**). In rice, 41 out of 103 *WRKY* genes exhibited variable expression patterns in response to chilling stress (Ramamoorthy et al., 2008). Moreover, we found three isoforms of *DREB* (dehydration-responsive element-binding, comp18139) transcription factor genes that were upregulated at 5°C (**Supplementary Table S1**). The important role of DREB transcription factor in plant stress signaling and activation of biotic and abiotic stress-responsive genes has been reported (Agarwal et al., 2006).

**FIGURE 5:**
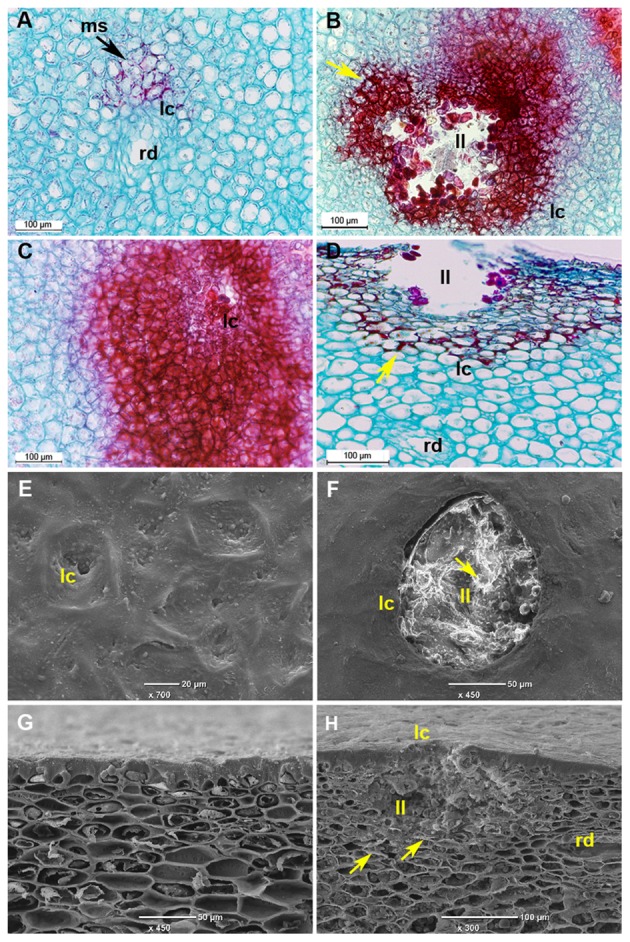
**Microscopic evaluation of discolored mango fruit lenticels after 19 days of cold storage. (A–D)** Histological sections of ‘Keitt’ mango peel stained with safranin and fast green after 3 weeks of cold storage. **(A)** Transverse section of peel of mango stored at 12°C, showing healthy lenticels. **(B,D)** Transverse and paradermal sections of mango stored at 5°C, respectively, showing discolored lenticels with phenolics accumulation (stained in red). **(C)** Transverse section of mango stored at 5°C showing higher accumulation of phenolics surrounding pitting. **(E–H)** Scanning electron microscopic images after 3 weeks of storage. **(E)** Top view and **(G)** cross section of healthy lenticels of mango fruit stored at 12°C. **(F)** Top view and **(H)** paradermal section of discolored lenticels of mango fruit stored at 5°C, respectively, showing lenticels’ deeper lumen (ll) with accumulation of dead cells. ms, modified stomata; ll, lenticel lumen; lc, lenticel; rd, resin duct; yellow arrow, phenolic accumulation.

This stress response signaling pathway (**Figure 4**) is known to activate the hypersensitive response and programmed cell death in response to pathogens (Kawasaki et al., 2005; Jones and Dangl, 2006). In our experiment, cell death was observed several days after activation of this signaling pathway in the lumen of discolored lenticels in response to long storage (19 days) at the suboptimal temperature of 5°C (**Figures 5B,D,F,H**).

### Cytological Changes during CI: Lenticel Discoloration

Several abiotic stress factors, such as sap from a cut pedicel (Loveys et al., 1992) and hot-water brushing during postharvest handling (Luria et al., 2014) have been observed to be triggers for lenticel discoloration in mango. In the present experiment, the sap was removed at the orchard and no hot-water brushing was applied. Black and red spots have also been characterized as CI symptoms (Pesis et al., 1997) and were observed here as well (**Figure 1**).

To characterize the morphology of discolored lenticels that appear after long storage at suboptimal temperature, we examined the transverse and paradermal orientation of non-discolored and discolored lenticels by histological staining with safranin and fast green and SEM observation. Light-microscopic observation of non-discolored lenticels showed formation of chilling-related lenticels from modified stomatal complexes (**Figure 5A**), as has been previously reported for other abiotic stress factors, such as sapburn (Du Plooy et al., 2009). These results demonstrated that expansion of lenticel discoloration results in pitting of the surrounding tissue. SEM analysis showed a 100–150-μm wide and deep hollow space in the lumen of blackened discolored lenticels (**Figures 5F,H**), which was opened by the accumulation of dead cells. This accumulation probably resulted from activation of the stress response-signal transduction that was observed after 2 days of storage at 5°C (**Figure 4**), which should induce programmed cell death. The consequent opening that occurs after a few weeks of storage could potentially allow invasion of pathogens into the fruit. Indeed, the level of CI and lenticel discoloration after cold storage was correlated with the incidence of peel decay after SL storage (**Figure 1B**; **Supplementary Figure S3**). On the other hand, the incidence stem-end rot was similar at all storage temperatures (**Supplementary Figure S3**). Increased susceptibility to decay following chilling has been previously observed in various fruits, and has been hypothesized to be related to weakening of the tissue and increased pathogen penetration (Lyons, 1973). Our results showed that peel decay follows lenticel damage, which is conducive to pathogen penetration through the enlarged openings.

### Chilling Stress-Activated Phenylpropanoid Pathway in Mango Peel

**FIGURE 6:**
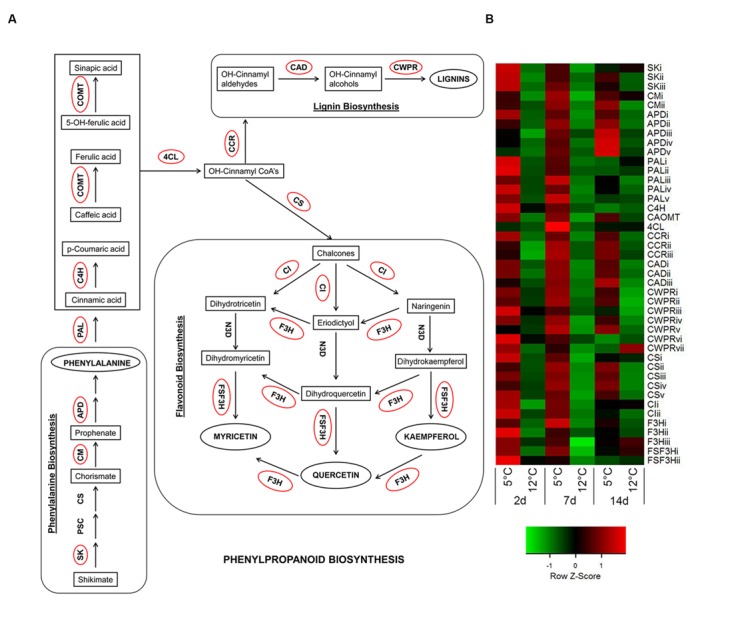
**Activation of phenylpropanoid-biosynthesis pathway in response to cold storage. (A)** Major connections of phenylalanine biosynthesis to phenylpropanoid-, flavonoid-, and lignin-biosynthesis pathways based on the KEGG pathway mapper. Genes circled in red are significantly upregulated during cold storage at 5°C. **(B)** Expression heat map of genes related to phenylpropanoid-biosynthesis pathway at two different storage temperatures (5 and 12°C) at different sampling times (2, 7, and 14 days). *Z*-scores represent rescaled log fold change values. Abbreviations, transcript identification and expression profile are described in **Supplementary Table S2**.

In addition to cell death, we observed that in the non-discolored samples, the lenticels do not stain intensively, indicating a lack of phenolics and lignin accumulation in the lenticel and its neighboring cells (**Figure 5A**). In contrast, the discolored lenticels were intensely stained in red color, indicating heavy accumulation of phenols and lignin in the cell wall and vacuole of tissue near the lenticel lumen (**Figures 5B,D**). Moreover, the pitted tissue surrounding the discolored lenticels was heavily stained, indicating intense accumulation of phenolics and lignin in this tissue as well (**Figure 5C**). The phenylpropanoid metabolic pathway is important for the synthesis of various phenolic compounds and lignin. A nearly complete gene set of the phenylpropanoid pathway was upregulated during cold storage, including genes for phenylalanine biosynthesis (**Figure 6**). Key genes of the pathway were significantly upregulated within 2 days of cold storage (**Figure 6**; **Supplementary Table S2**).

The phenylpropanoid pathway is commonly upregulated in response to various abiotic stresses, leading to accumulation of flavonoids and lignins (Dixon and Paiva, 1995). Lignin is a polymer of phenylpropanoid that contributes substantially to cell wall firmness and stability in response to biotic and abiotic stresses (Vogt, 2010). Plants change their lignin content and composition in response to various stresses (Moura et al., 2010). It has been well reported that in response to low temperature, the activities of lignin-synthesis enzymes change in plants (Hausman et al., 2000; Janas et al., 2000), and that lignin biosynthesis genes are upregulated in response to cold stress in many plants (Moura et al., 2010; Zhu et al., 2013). Similarly, in our transcriptomic data, genes for the synthesis of all lignin monomers were upregulated at 5°C (**Figure 6**; **Supplementary Table S2**).

Phenylpropanoid pathway genes have been reported to be upregulated during suboptimal cold storage of blood oranges and table grapes leading to accumulation of flavonoids and anthocyanins (Sanchez-Ballesta et al., 2007; Crifò et al., 2011). In mango fruit, we found significant upregulation of flavonoid biosynthesis-related genes for the synthesis of quercetin, myricetin, tricetin, and kaempferol at 5°C (**Figure 6**), whereas the anthocyanin biosynthesis genes were not upregulated and no anthocyanin accumulation was found in response to chilling (data not shown). Thus, phenolics accumulation in the discolored lenticel (**Figure 5**) was probably due to activation of the phenylpropanoid pathway (**Figure 6**).

### Transportation of Phenols from the Resin Ducts to Discolored Lenticels

**FIGURE 7:**
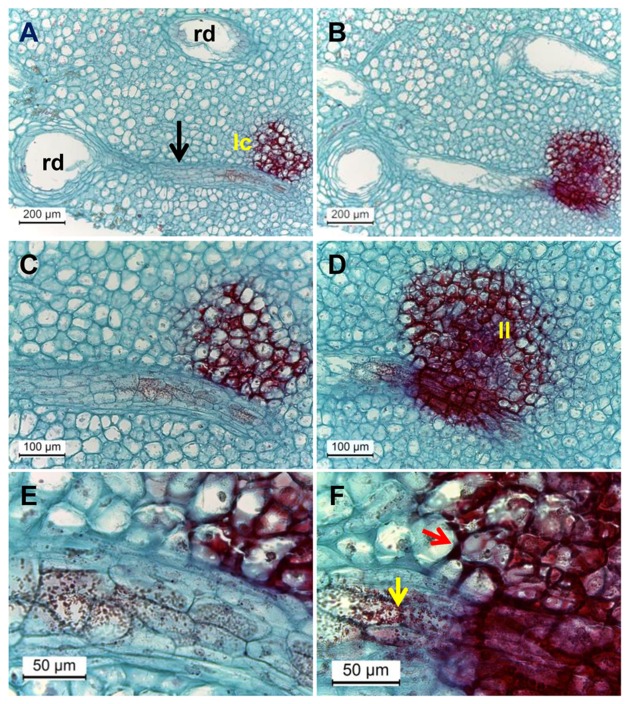
**Microscopic evaluation of resin duct contact with discolored lenticels in surface and deeper transverse histological sections of mango peel stained by safranin and fast-green after 3 weeks of cold storage. (A–F)** Transverse sections of mango stored at 5°C, observed at various magnifications (**A**, **B**, magnification ×100; **C**, **D**, magnification ×200; **E, F**, magnification ×400) showing discolored lenticels with phenolic accumulation (stained in red) and lenticel connection to resin duct. Dense red-colored granules are seen inside the resin duct. **(B**,**D,F)** Are surface cuts and **(A**,**C,E)** are deeper cuts. rd, resin duct; lc, lenticel; black arrow, zone of mesophyll cells seen between rd and lc; yellow and red arrows, phenolic transport and accumulation, respectively.

Are phenolic compounds synthesized locally in the discolored lenticel area or are they transported? Light microscopic observation of deep sections of discolored lenticels frequently showed direct contact between the resin ducts and discolored lenticels connected by a distinct zone of mesophyll cells (**Figure 7A**). Safranin-stained phenolic granules were seen throughout this zone transporting phenols from the resin duct to lenticels (**Figure 7C**). The granules appeared half-way across the connective zone, moving toward the lenticels (**Figures 7C,E**). Penetration and accumulation of phenolics in the cell wall of mesophyll cells was seen in the deep sections (**Figure 7F**). The phenolics in the cell wall were organized in a half circle facing the lenticels (**Figure 7F**). Phenolics accumulation was also observed in the vacuoles and cytoplasmic region of sub-lenticel cells (**Figure 7D**). These observations extend those made in previous studies showing the deposition of phenolics in cell walls of sub-lenticel cells (Du Plooy et al., 2006). Those phenolic compounds might be gallic and ferulic acid derivatives and cinnamic acid (Du Plooy et al., 2009), resulting from activation of the phenylpropanoid pathway (**Figure 6**). At a later stage, cells lining the lenticel lumen were fully laden with dense phenolics, staining dark red (**Figures 5B,D** and **7B,D,F**), possibly due to phenolics oxidation which darkens the lenticels (Du Plooy et al., 2009). Phenolic compounds and reinforced cell wall normally form a protective barrier that resists pathogen attack (Dixon and Paiva, 1995). However, at suboptimal temperature storage (5°C), these cells were loosened from neighboring tissue and formed a wider hollow space of lenticel lumen (**Figures 5B,D,F**). Such openings increase susceptibility to pathogenic fungal colonization (**Supplementary Figure S3**).

The release of terpenes from resin ducts causes endomembrane collapse in sub-lenticel cells (Bezuidenhout et al., 2005). Thus, the vacuolar phenols contact the cell wall polyphenol oxidase (PPO), resulting in lenticel discoloration. In contrast, Du Plooy et al. (2006) observed intact cellular organization, even in the darkened lenticels, and suggested that the PPO was not released and that lenticel discoloration is due to the accumulation of phenolics inside the vacuoles. Our transcriptomic data revealed that PPO (*PPO1*, *PPO2*, *PPO3*) expression were maintained at maximum level until 7 days of cold stress at 5°C, whereas all *PPO*s were downregulated under 12°C storage (**Supplementary Figure S4**). Thus, the expression level of *PPO*s was 3.1-fold and 3.6-fold higher at 5°C vs. 12°C after 2 and 7 days of cold storage, respectively. In support of *PPO* upregulation, we also observed pulp browning beneath the subcutaneous layer in a few fruit stored at 5°C (**Supplementary Figure S5**), which we characterized as severe CI. This suggests that PPOs cause mango pulp browning during chilling stress. However, whether this plays a role in lenticel discoloration is unclear.

### Lipid Peroxidation as Established by IVIS and MDA Analysis

**FIGURE 8:**
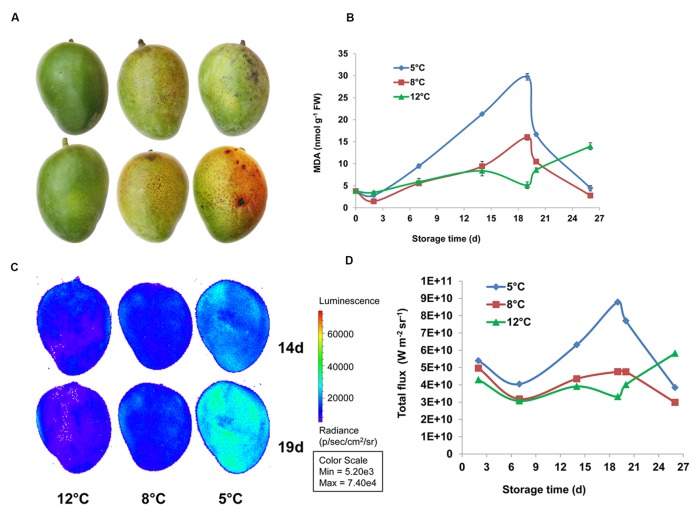
**Lipid peroxidation observed in mango during cold storage. (A)** Representative pictures of fruit stored at 12°C (left panel), 8°C (middle panel), and 5°C (right panel) for 19 days. **(B)** Malondialdehyde (MDA) concentration in mango fruit peel stored for 19 days at 12°C (green), 8°C (red), or 5°C (blue) and transferred to 20°C for the remaining 8 days. **(C)** Representative pictures of luminescing peroxide lipids in fruit stored at 12°C (left panel), 8°C (middle panel), 5°C (right panel) for 14 days (upper panel) or 19 days (lower panel). **(D)** Quantification of luminescence (as presented in **C**) in mango fruit stored for 19 days at 12°C (green), 8°C (red), or 5°C (blue) and transferred to 20°C for the remaining 8 days, values are presented as W m^-2^ sr^-1^.

Chilling-induced oxidative stress is a well-known cause of membrane lipid peroxidation (Sagi and Fluhr, 2006; Miller et al., 2008). MDA is a peroxide lipid derivative. The effect of chilling stress on peroxidation of membrane lipids was determined in fruit during cold storage at 12, 8, and 5°C and further SL storage by quantifying MDA accumulation. MDA started accumulating in the peel after 7 and 14 days of cold storage at 5 and 8°C, respectively. Maximum MDA accumulation was observed after 19 days of cold storage at 5 and 8°C, to 6.9-fold and 3.2-fold the level at harvest, respectively (**Figure 8B**). After SL storage, MDA decreased in fruit from 5 and 8°C storage back to harvest levels. While MDA was not accumulated in fruit that were stored at 12°C and only after the transfer to SL it started to accumulate, possibly as a result of over-ripening (**Figure 8B**). Our results indicated that chilling stress induces membrane disruption due to lipid peroxidation. Malondialdehyde accumulation in mango was previously observed in cv. Tainong peel after 7 days of cold storage at 4°C (Wang et al., 2008).

Luminescence measurement of lipid peroxidation in mango cv. Shelly and avocado fruit during cold storage has been optimized as a non-destructive, efficient method (Sivankalyani et al., 2015, 2016). Here, we show for the first time that MDA accumulation in fruit peel correlates well with luminescence of peroxide lipids from whole fruit detected by IVIS (**Figures 8C,D**). Thus, luminescence and MDA measurements indicated that fruit stored at 12°C has only minor lipid peroxidation, fruit that stored at 8°C has a moderate level, and fruit that stored at 5°C has maximum lipid peroxidation, from 14 to 19 days of storage (**Figure 8**).

### Activation of Lipid-Related Metabolism in Mango Peel in Response to Chilling Stress

As already noted, lipid peroxidation was characterized long ago as one of the key responses to chilling (Wise and Naylor, 1987). Mango fruit also responded with lipid peroxidation to suboptimal temperature storage (**Figure 8**). Lipid-related metabolic pathways such as glycerophospholipid metabolism and oxidation of α-linolenic acid were activated in mango fruit in response to chilling stress. The α-linolenic acid metabolic pathway, which leads to oxidation of lipids, was upregulated in our transcriptomic data in response to storage at 5°C. Specifically, four isoforms of lipoxygenase (*LOX*), and three genes of allene oxide synthase (*AOS*), allene oxide cyclase (*AOC*), and 12-oxophytodienoate reductase (*12-ODR*) were upregulated (**Supplementary Table S2**). Interestingly, this metabolic pathway not only oxidizes lipid but also drives the pathway for methyl-jasmonate synthesis. Methyl-jasmonate is known to play a main role in protection against chilling in various fruit, including mango (Bell and Mullet, 1993; Gonzalez-Aguilar et al., 2000; Sivankalyani et al., 2015).

**FIGURE 9:**
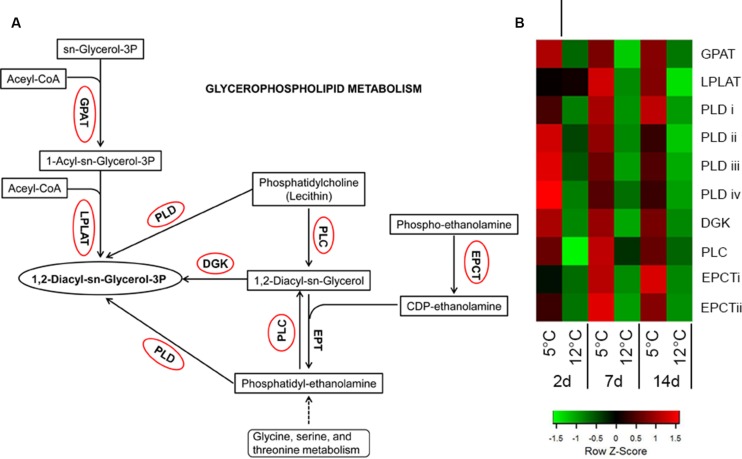
**Activation of glycerophospholipid metabolism-related genes in response to cold storage. (A)** Glycerophospholipid metabolism based on the KEGG pathway mapper. Genes circled in red are significantly upregulated during cold storage at 5°C. **(B)** Expression heat map of genes related to glycerophospholipid metabolism at two different storage temperatures (5 and 12°C) at different sampling times (2, 7, and 14 days). *Z*-scores represent rescaled log fold change values. Abbreviations, transcript identification and expression profile are described in **Supplementary Table S2**.

Glycerophospholipid metabolism is an important pathway that was significantly activated during cold stress at 5°C (**Figure 9**; **Supplementary Table S2**). It results in the synthesis of phosphatidic acid (PA), an important signaling molecule produced in plants in response to stress (Testerink and Munnik, 2005). Key genes of PA synthesis, such as phospholipases D and C (*PLD* and *PLC*, comp18965, comp24723, comp25980, comp17158, comp37611) and diacylglycerol kinase (*DGK*, comp20642), were upregulated in this pathway (**Figure 9**). Phospholipases are recognized as key factors in plant responses to biotic and abiotic stresses (Xue et al., 2007). PLC and PLD hydrolyze phospholipids, particularly the phosphatidylcholine (PC) and phosphatidylethanolamine (PE) components of biological membranes, and produce important signaling molecules, such as PA, oxylipins and jasmonate (Laxalt and Munnik, 2002; Xue et al., 2007). Similarly, cold storage of mango upregulated isoforms of *PLD* and *PLC* within 2 days (**Figure 9**; **Supplementary Table S2**). PLC cleaves PC/PE into *DGK* and further into PA by DGK. A mild increase in DGK was found in response to cold storage (**Figure 9**; **Supplementary Table S2**). These signaling molecules modulate the activity of MAPKs, proteins involved in membrane-trafficking, Ca^2+^-signaling and the oxidative burst (Laxalt and Munnik, 2002; Xue et al., 2007). Indeed, mango fruit responded to suboptimal temperature storage by activation of MAPK, Ca^2+^-signaling and NADPH oxidase (**Figure 4**).

### CI Induction of Selected Pathways

#### Sugar Metabolism

Cold stress triggers the accumulation of soluble sugars in potato (Oufir et al., 2008), orange (Rapisarda et al., 2001), and mandarin (Manolopoulou-Lambrinou and Papadopoulou, 1995). Accumulation of simple sugars is likely to contribute to the stabilization of membrane phospholipids, thereby protecting the membranes against freeze damage (Thomashow, 1999). Accumulation of simple sugars in response to cold stress has been proposed to be due to *de novo* synthesis of organic acids through gluconeogenesis (Echeverria and Valich, 1989) or via UDP-D-glucose (Zhu et al., 2011). Our data showed that chilling stress activates sugar metabolism in mango fruit. Sugar metabolism involves the synthesis of osmolytes such as sucrose, trehalose, raffinose and stachyose via UDP-glucose. Mango fruit stored at suboptimal temperature upregulated the genes involved in sugar metabolism, i.e., those encoding sucrose synthase, six isoforms of T6PS for trehalose synthesis, α-amylase and β-amylase (**Supplementary Figure S6**; **Supplementary Table S2**).

#### Hormone Signal Transduction

Gibberellin (GA), ethylene, jasmonic acid (JA), and salicylic acid (SA) play substantial direct or indirect roles in plant responses to abiotic stress (Peleg and Blumwald, 2011). Here, several genes involved in GA-, ABA-, ethylene-, JA-, and SA-mediated signaling were upregulated in mango fruit in response to storage at 5°C (**Supplementary Table S2**). *Arabidopsis* DELLA transcription factors enable plants to respond to GA. DELLA is induced in response to cold stress and it is known to increase plant tolerance to freezing (Achard et al., 2008). Mango *DELLA* (comp13877) was found to be upregulated 4.5-fold after 2 days of storage at 5°C. In *Arabidopsis*, exogenous application of GA increases the expression of genes encoding NPR1, which is involved in SA biosynthesis and action (Alonso-Ramires et al., 2009). In mango, two isoforms of *NPR1* (comp20774, comp2117) were upregulated in response to cold. SA and JA are known to be correlated with increased tolerance of fruit to suboptimal cold storage (Gonzalez-Aguilar et al., 2000; Cao et al., 2009). Our transcriptomic analysis showed upregulation of the JA-responsive gene *JAZ* (comp20061, comp7092, comp6212) in response to cold. JA and ethylene act together as a common stress response (Alkan and Fortes, 2015). The ethylene response increases mango fruit tolerance to chilling (Lederman et al., 1997). In our transcriptome data, the following ethylene-responsive genes were upregulated: ethylene-insensitive 3 (*EIN3*, comp17311, comp19306), EIN3-binding f-box (*EBF1/2*, comp15373, comp29914), and ethylene-responsive transcription factor (*ERF1/2*, comp6324, comp20003). We suggest that upregulation of hormone-related genes (GA, JA, SA, and ethylene) is the fruit’s natural response to extreme cold storage. A similar response is likely to help the fruit coping with shorter or more moderate cold stress.

#### Processing of Endoplasmic Reticulum (ER) Proteins

Under adverse environmental conditions, misfolded or unfolded proteins accumulate in the ER and cause ER stress (Liu and Howell, 2010). Indeed, transcripts related to protein processing in the ER were found to be upregulated, including the ER-associated degradation (ERAD) system of misfolded/unfolded proteins (**Supplementary Table S2**). Genes encoding components of the protein-folding machinery and transport: *Bip* (comp24844) and *SAR1* (comp21135, comp21200, comp21264), were upregulated. BIP is an ER protein related to stress, including cold (Anderson et al., 1994), which binds with folding intermediates and results in misaggregation of proteins within the ER (Liu and Howell, 2010). SAR1 is involved in vesicle transport of correctly folded proteins from the ER to the Golgi. In all, 36 genes related to the ERAD system and its ubiquitin ligase complex were significantly upregulated in mango fruit in response to suboptimal cold storage, mainly after 7 and 14 days (**Supplementary Table S2**). These results indicated that protein processing in the ER has a major role in the fruit’s response to chilling.

## Conclusion

**FIGURE 10:**
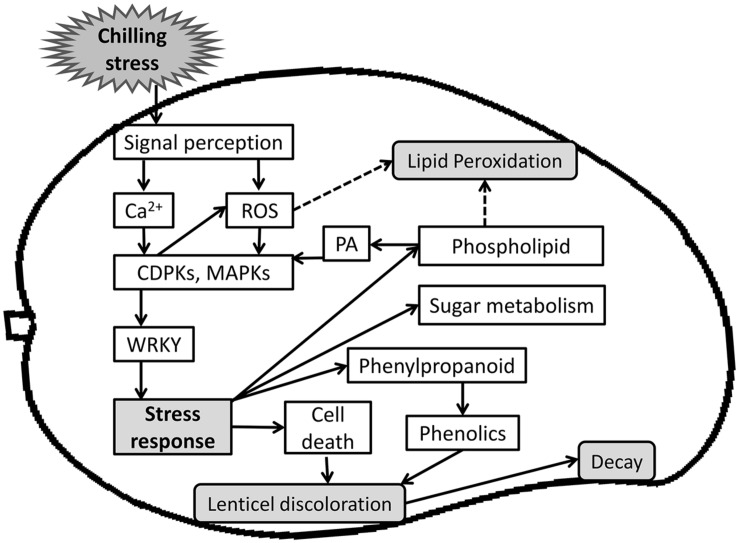
**A Scheme summarizing events of mango fruit response to storage at sub-optimal temperature.** Upon storage at sub-optimal temperature, fruit sense the chilling stress and initiate cold stress signal transduction pathway. A cascade of signaling molecules (Ca^2+^, ROS) act as secondary messengers and activates several kinases (CDPK, MAPK), which activates transcription factors (WRKY) and further activate stress response. Ultimately, a wide range of stress responses were activated, such as phenylpropanoid pathway that produces phenols, which is transported from the resin-duct to the lenticel, and cell death that accumulate in the same lenticel lumen and is tightly correlated to the later increase in decay. Chilling stress response also activates sugar metabolism for osmolytes synthesis and phospholipid metabolism that leads to synthesis of PA, which is known to activate CDPK and MAPK signaling and stress response.

Cold storage is the best known technique to extend postharvest fruit life. However, CI limits the application of cold treatment to tropical fruit such as mango. In this work, we elucidated and characterized the molecular basis of mango fruit’s response to chilling during postharvest cold storage. Two days after storage at suboptimal temperature, mango fruit upregulated transcripts of membrane receptors that induce oxidative stress and other signaling molecules such as calcium and MAPK, to further activate the downstream genes of the fruit response to chilling (**Figure 10**). We showed that red spots and black spots of discolored lenticels are CI symptoms which, upon expansion, result in pitting. Lenticel discoloration is probably due to the accumulation of oxidized phenols that are transported from the resin ducts and are the result of phenylpropanoid-pathway activation, as shown by the transcriptome data. A few weeks after suboptimal storage, we observed accumulation of dead cells in the lumen of the discolored lenticels, which correlated with the upregulation of the stress response-related transcripts observed 2 days after cold storage (**Figure 10**). The increase in red and black spots after storage at suboptimal temperature was correlated to the accumulation of general decay on the peel after further SL storage, suggesting that these openings in the fruit cuticle are preferred sites for pathogenic fungus penetration and attack. In addition, CI of mango fruit was accompanied by a direct increase in lipid peroxidation and concomitant upregulation of transcripts related to the α-linolenic acid oxidative pathway and glycerophospholipid metabolism (**Figure 10**). This extensive characterization of mango fruit’s natural response to storage at suboptimal temperature should facilitate future research and provide a basis for improving fruit tolerance to cold storage.

## Availability of data and materials

All data analyzed in this manuscript were deposited in GenBank under accession number SRP066658.

## Author Contributions

VS carried out the experiment and data analysis and prepared the manuscript. NS analyzed the bioinformatics data and drafted the manuscript. OF carried out the experiment and participated in the data analysis. HZ conducted the microscope analyses and drafted the manuscript. DM carried out the experiment and participated in data analysis. NA supervised the study, the experiments, and the data analysis and prepared the manuscript. All authors read and approved the final manuscript.

## Conflict of Interest Statement

The authors declare that the research was conducted in the absence of any commercial or financial relationships that could be construed as a potential conflict of interest.

## Footnotes

1 http://www.genome.jp/kegg/

2 http://www.genome.jp/kegg/tool/map_pathway2.html

## Supplementary Material

FIGURE S1 **Physiological parameters of ‘Keitt’ mango fruit ripening.** Ripening-related parameters were quantified after 19 days of cold storage (5, 8, 10, or 12°C; black column) and after 7 additional days at 20°C (white column). **(A)** Firmness in Newton. **(B)** Percent of total soluble sugars (TSS). **(C)** Fruit peel color (Hue). **(D)** Percent citric acid.

FIGURE S2 **Significantly overrepresented GO terms for mango ‘Keitt’ in response to cold stress.** Overrepresented GO terms in response to cold stress are shown as –log10 of the FDR corrected *p*-value in **(A)** cluster 2, **(B)** cluster 4, and **(C)** cluster 5. The GO terms are separated into biological process and molecular function.

FIGURE S3 **Percentage of decay in mango fruit after storage. (A)** Percentage of general decay on the peel, and **(B)** percentage of stem-end rot (SER) in ‘Keitt’ mango fruit stored at cold temperature (5, 8, 10, or 12°C) for 3 weeks and for 7 days at 20°C (blue column), and for an additional 3 days at 20°C (red column).

FIGURE S4 **Relative expression of polyphenol oxidase in response to cold stress.** Relative expression of three polyphenol oxidases represented by RPKM values at harvest and after storage at 12 or 5°C for 2, 7, and 14 days. Presented are average ±SE.

FIGURE S5 **Internal browning of ‘Keitt’ mango fruit in response to cold stress.** Representative pictures of ‘Keitt’ mango fruit internal browning after cold storage for 19 days followed by 7 days at 20°C. **(A)** Cold storage at 12°C. **(B,C)** Cold storage at 5°C. Arrow indicates severe internal browning.

FIGURE S6 **Activation of starch, sucrose and galactose metabolism-related genes in response to cold storage. (A)** Starch, sucrose, and galactose metabolism based on the KEGG pathway mapper. Genes circled in red are significantly upregulated during cold storage at 5°C. **(B)** Expression heat maps of genes related to starch, sucrose and galactose metabolism at two different storage temperatures (5 and 12°C) at different sampling times (2, 7, and 14 days). Abbreviations, transcript identification and expression profile are described in **Supplementary Table S2**.

TABLE S1 **Mango ‘Keitt’ transcript expression profile at harvest and at 2, 7, and 14 days of cold storage.** Shown are: Transcript ID (comp number), gene description (based on Blast2GO annotation), KEGG number, GO description, enzyme code (EC), InterProScan, RPKM of different biological replicates, cluster. Also presented are fold change, *p*-value and FDR (adjusted *p*-value) of different comparisons.

TABLE S2 **Abbreviations and transcript identification presented and divided according to pathways and the Figures in which they appear.** Transcript name abbreviation, Transcript ID (comp number), gene description (based on Blast2GO annotation), KEGG number, GO description, enzyme code (EC), InterProScan, RPKM of different biological replicates, cluster. Also presented are: fold change, *p*-value, FDR (adjusted *p*-value) of different comparisons. Last columns present significant induction of the gene at 5°C after 2, 7, and 14 days compared to 12°C cold storage.
