# Supplementary figures and images for: Transcriptome Dynamics in Mango Fruit Peel Reveals Mechanisms of Chilling Stress
Source: Front Plant Sci. 2016 Oct 20;7:1579. doi: 10.3389/fpls.2016.01579 (PMC5072284; doi:10.3389/fpls.2016.01579)

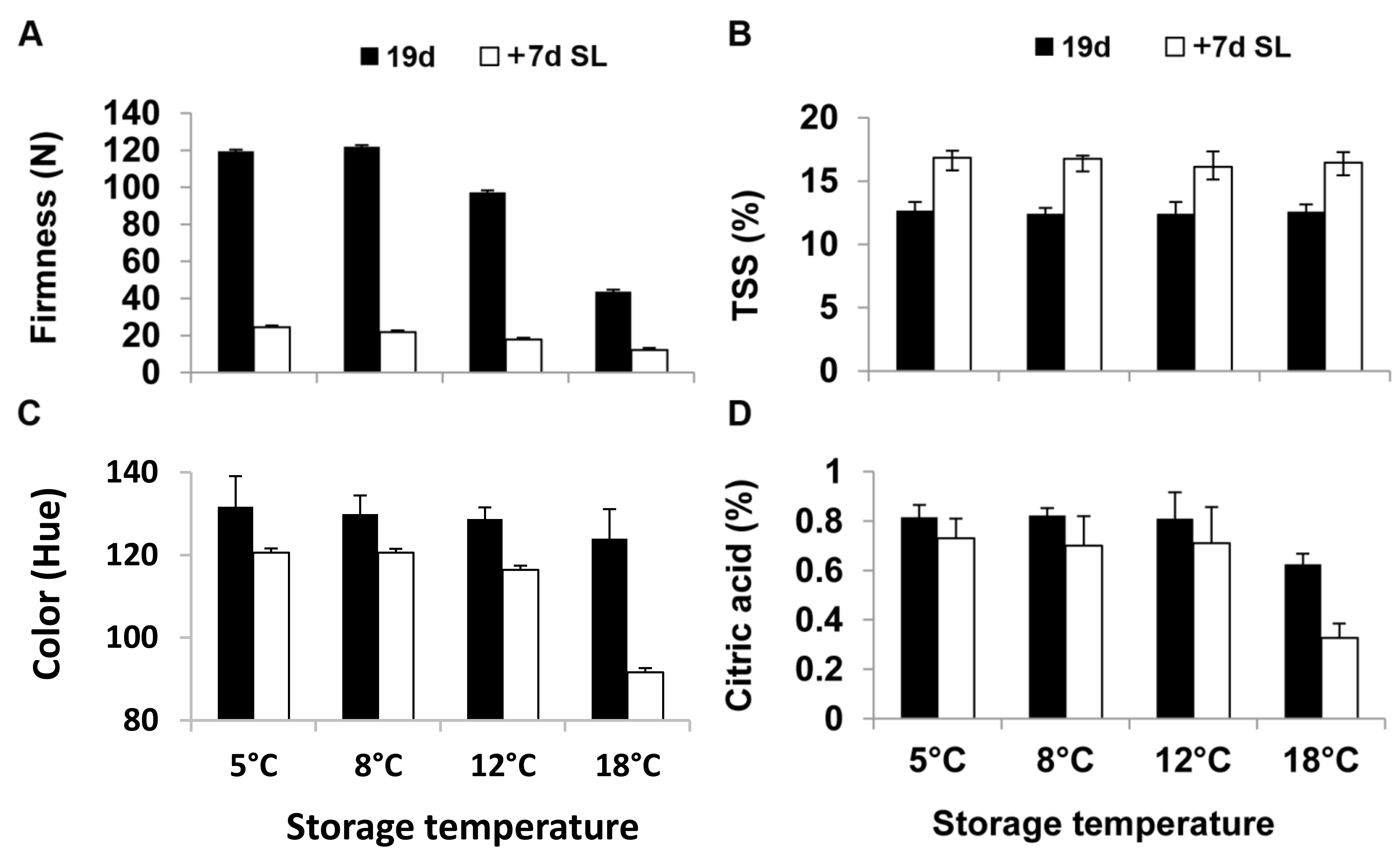

Supplement: FIGURE S1 — Physiological parameters of ‘Keitt’ mango fruit ripening. Ripening-related parameters were quantified after 19 days of cold storage (5, 8, 10, or 12°C; black column) and after 7 additional days at 20°C (white column). (A) Firmness in Newton. (B) Percent of total soluble sugars (TSS). (C) Fruit peel color (Hue). (D) Percent citric acid. [file Image_1.TIF]

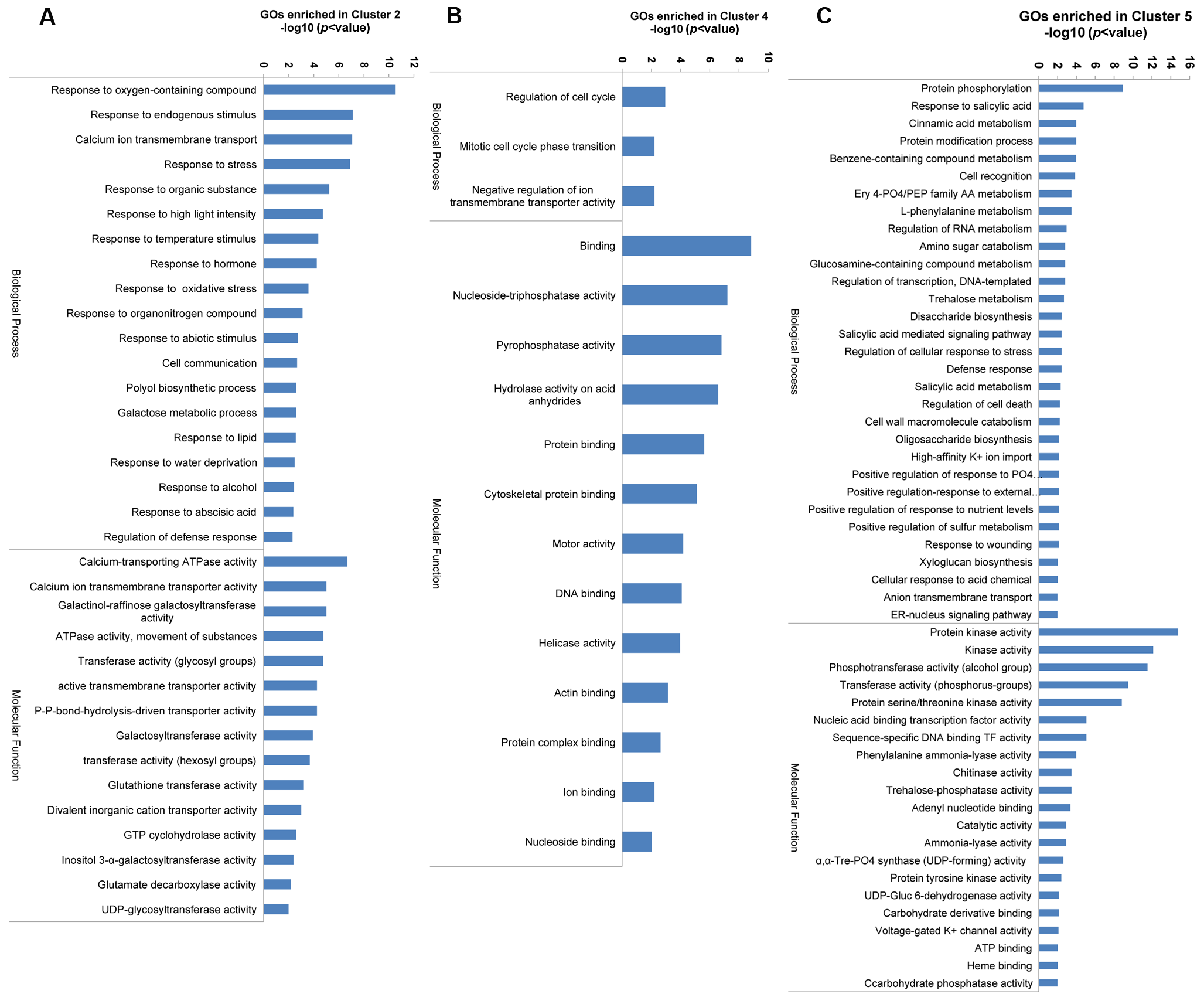

Supplement: FIGURE S2 — Significantly overrepresented GO terms for mango ‘Keitt’ in response to cold stress. Overrepresented GO terms in response to cold stress are shown as –log10 of the FDR corrected p-value in (A) cluster 2, (B) cluster 4, and (C) cluster 5. The GO terms are separated into biological process and molecular function. [file Image_2.TIF]

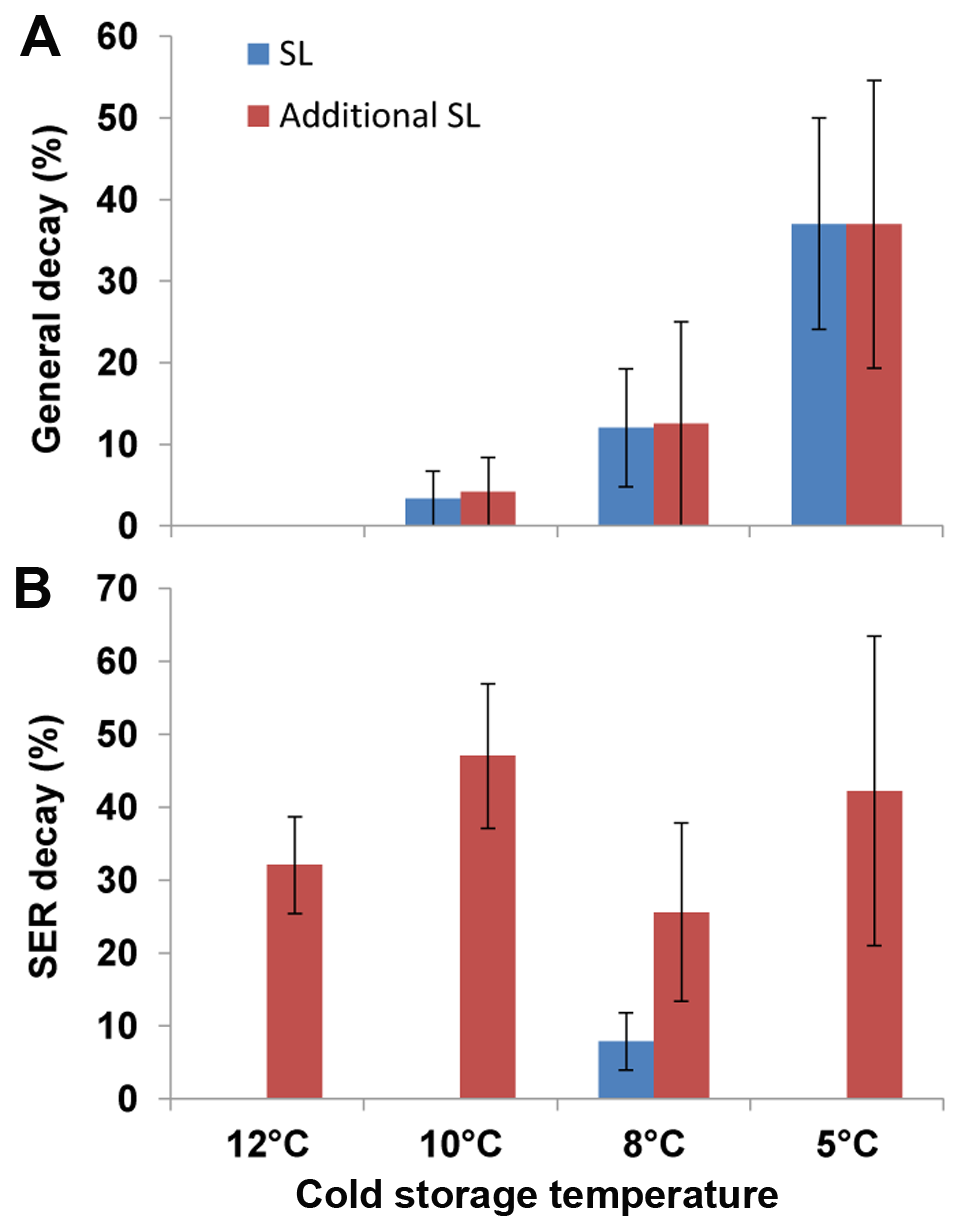

Supplement: FIGURE S3 — Percentage of decay in mango fruit after storage. (A) Percentage of general decay on the peel, and (B) percentage of stem-end rot (SER) in ‘Keitt’ mango fruit stored at cold temperature (5, 8, 10, or 12°C) for 3 weeks and for 7 days at 20°C (blue column), and for an additional 3 days at 20°C (red column). [file Image_3.TIF]

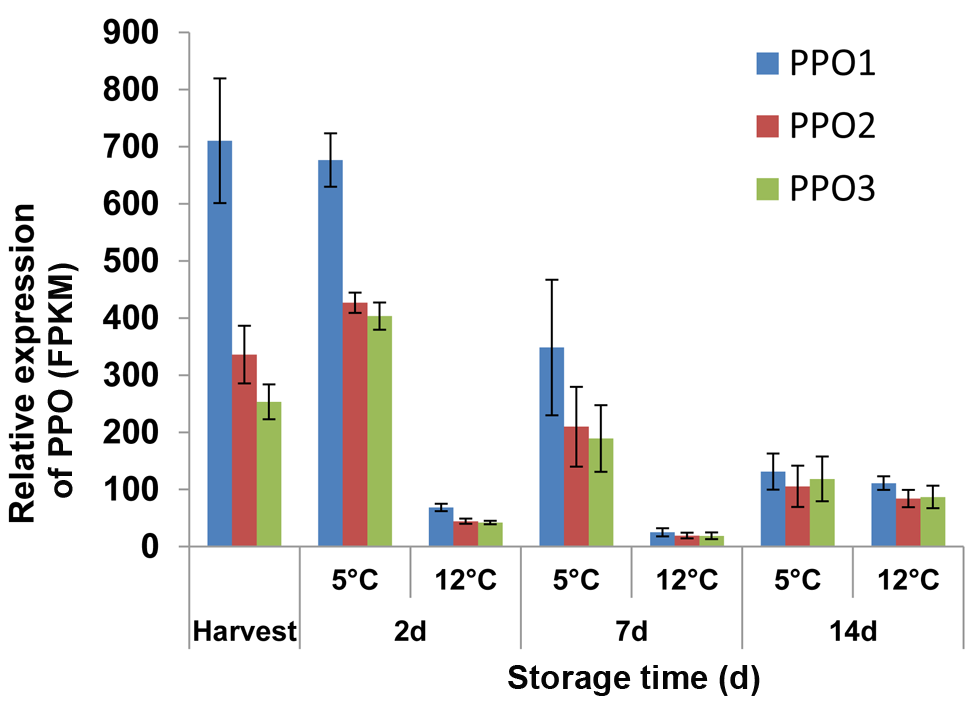

Supplement: FIGURE S4 — Relative expression of polyphenol oxidase in response to cold stress. Relative expression of three polyphenol oxidases represented by RPKM values at harvest and after storage at 12 or 5°C for 2, 7, and 14 days. Presented are average ±SE. [file Image_4.TIF]

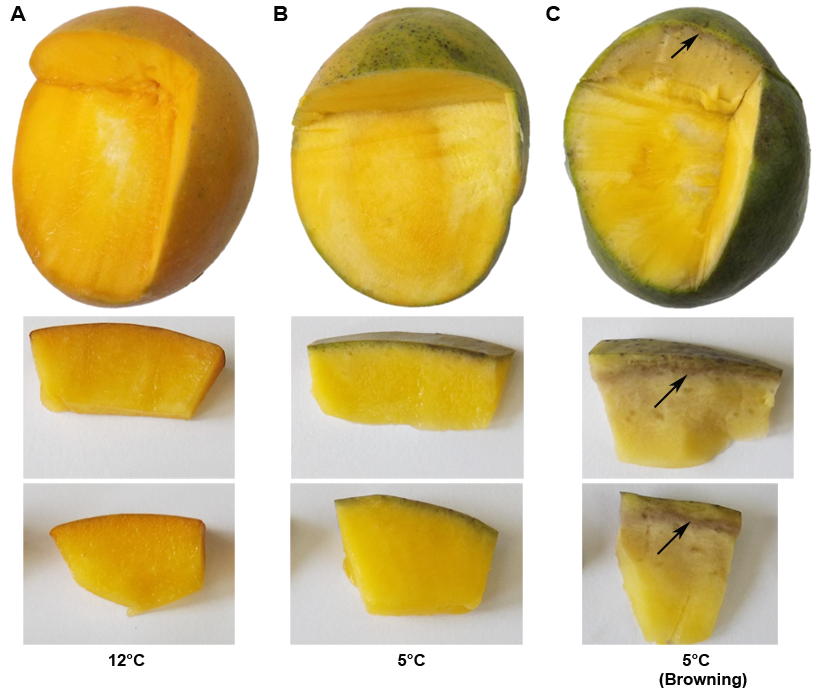

Supplement: FIGURE S5 — Internal browning of ‘Keitt’ mango fruit in response to cold stress. Representative pictures of ‘Keitt’ mango fruit internal browning after cold storage for 19 days followed by 7 days at 20°C. (A) Cold storage at 12°C. (B,C) Cold storage at 5°C. Arrow indicates severe internal browning. [file Image_5.TIF]

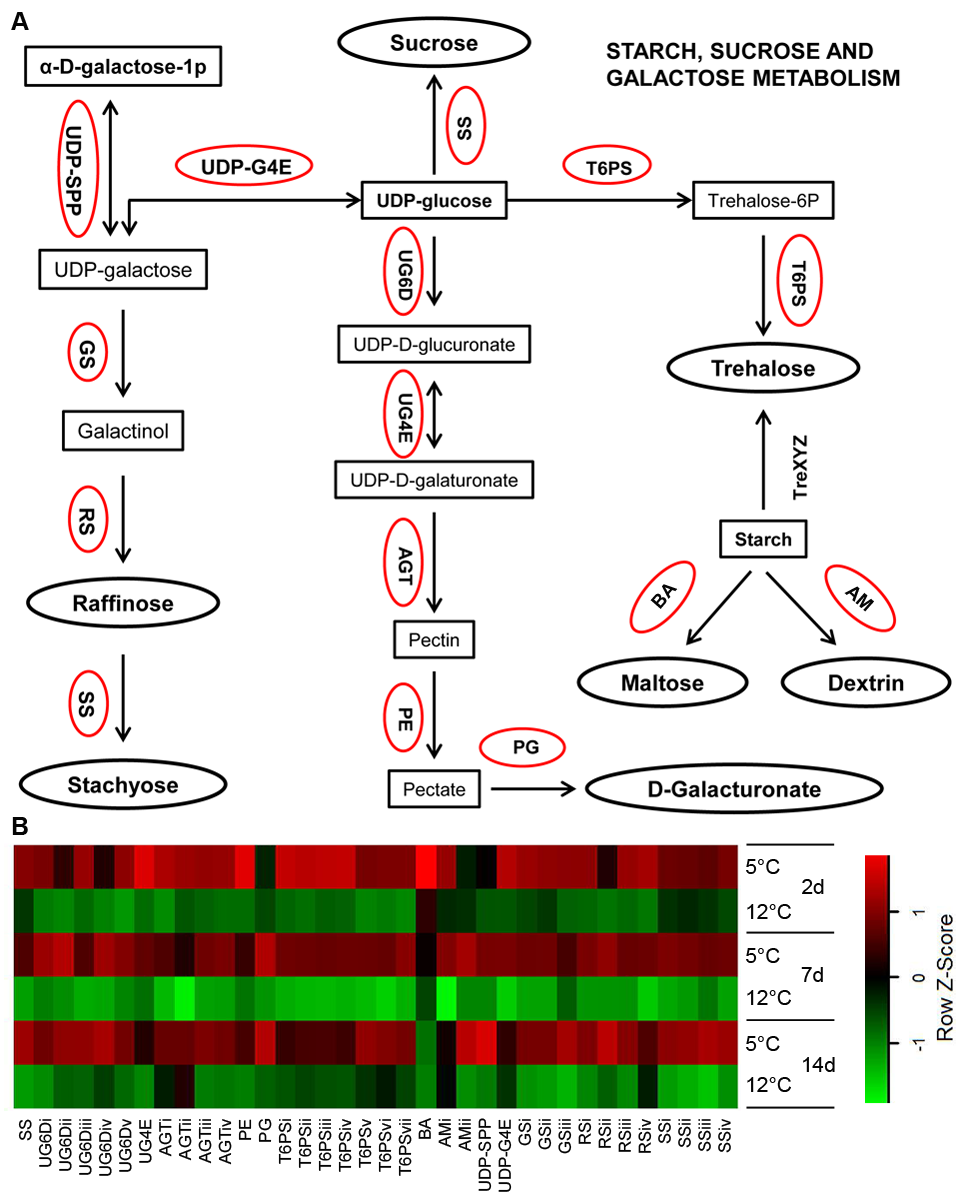

Supplement: FIGURE S6 — Activation of starch, sucrose and galactose metabolism-related genes in response to cold storage. (A) Starch, sucrose, and galactose metabolism based on the KEGG pathway mapper. Genes circled in red are significantly upregulated during cold storage at 5°C. (B) Expression heat maps of genes related to starch, sucrose and galactose metabolism at two different storage temperatures (5 and 12°C) at different sampling times (2, 7, and 14 days). Abbreviations, transcript identification and expression profile are described in Supplementary Table S2. [file Image_6.TIF]
